# Association between non-insulin-based insulin resistance indices and cardiovascular events in patients undergoing percutaneous coronary intervention: a retrospective study

**DOI:** 10.1186/s12933-023-01898-1

**Published:** 2023-06-29

**Authors:** Zenglei Zhang, Lin Zhao, Yiting Lu, Xu Meng, Xianliang Zhou

**Affiliations:** Department of Cardiology, Fuwai Hospital, National Center for Cardiovascular Diseases, Chinese Academy of Medical Sciences and Peking Union Medical College, No.167, Beilishi Road, Xicheng District, Beijing, 100037 China

**Keywords:** Coronary artery disease, Insulin resistance, Cardiovascular outcomes, Percutaneous coronary intervention, Metabolic score for insulin resistance

## Abstract

**Background:**

Insulin resistance (IR) has been confirmed that getting involved in the pathophysiological process of cardiovascular diseases (CVD). Recently, increasing evidence suggests metabolic score for insulin resistance (METS-IR), triglyceride to high-density lipoprotein cholesterol (TG/HDL-C) ratio, triglyceride and glucose (TyG) index, triglyceride glucose-body mass (TyG-BMI) index are simple and reliable surrogates for IR. However, their abilities in predicting cardiovascular outcomes in patients undergoing percutaneous coronary intervention (PCI) are not well explored. Therefore, this study aimed to investigate the association and evaluate the predictive performance of each index.

**Methods:**

A total of 2533 consecutive participants undergoing PCI were included in this study, and the data from 1461 patients were used to determine the correlation of these non-insulin-based IR indices with major adverse cardiac and cerebrovascular events (MACCEs) *via* performing the multivariate logistic models and restricted cubic splines (RCS).

**Results:**

During a median of 29.8 months follow-up, 195 cases of 1461 patients experienced incident MACCEs. In the overall population, both univariate and multivariate logistic regression analyses indicated no statistically significant connection between these IR indices and MACCEs. Subgroup analyses revealed significant interactions between age subgroups and TyG-BMI index, as well as METS-IR, and between sex subgroups and TyG index. In elderly patients, per 1.0-SD increment in TyG-BMI index and METS-IR had a significant association with MACCEs, with odds ratios (ORs) [95% confidence interval (CI)] of 1.24 (1.02–1.50) and 1.27 (1.04–1.56), respectively (both *P* < 0.05). Moreover, in female patients, all the IR indices showed significant associations with MACCEs. Multivariable-adjusted RCS curves demonstrated a linear relationship between METS-IR and MACCEs in elderly and female patients, respectively. However, all the IR indices failed to enhance the predictive performance of the basic risk model for MACCEs.

**Conclusion:**

All the four IR indices showed a significant association with MACCEs in female individuals, whereas only TyG-BMI index and METS-IR showed associations in elderly patients. Although the inclusion of these IR indices did not improve the predictive power of basic risk model in either female or elderly patients, METS-IR appears to be the most promising index for secondary prevention of MACCEs and risk stratification in patients undergoing PCI.

**Supplementary Information:**

The online version contains supplementary material available at 10.1186/s12933-023-01898-1.

## Introduction

Coronary artery disease (CAD) has been the first major cause of death worldwide, posing a heavy burden on public health and healthcare costs [1, 2]. Patients undergoing PCI for CAD should receive guideline-directed medical treatment as part of secondary prevention strategies to improve the clinical outcomes [3–5]. Although the excellent performance of drug-eluting stents (DES) in reducing restenosis and advancement in medical care have noticeably improved the outcomes, some individuals, like with hypertension or diabetes mellitus (DM), still face a high risk of recurrences of cardiovascular events [6, 7]. Therefore, early and optimized risk stratification has far-reaching significance in improving the secondary prevention of patients who undergo PCI.

Insulin resistance (IR), which refers to the diminished or impaired insulin sensitivity of target organs or tissues shown as impairments in absorbing and oxidizing the glucose, has been confirmed as an important risk factor in pathogenesis of DM and CVD [8, 9]. The underlying mechanisms may account for the role of IR in endothelial impairment [8], ectopic angiotensinogen production [10], and inappropriate activation of renin-angiotensin-aldosterone system (RAAS) [11]. Although the hyperinsulinemic-euglycemic clamp is regarded as the gold-standard method for assessing IR [12], its clinical application is limited due to cost and complexity. Fortunately, several alternative measures of IR have been established that are more convenient and still valid, such as METS-IR [13], TG/HDL-C [14, 15], TyG index [16], and TyG-BMI index [17]. Previous evidence has shown that these four IR indices are significantly associated with various CVD risk factors, including DM, metabolic syndrome, and arterial stiffness progression [10, 18–21]. Moreover, accumulating evidence suggests that these IR indices can predict the presence and severity of CAD, as well as adverse cardiovascular outcomes like stroke, acute myocardial infarction (AMI), and mortality [22–25].

However, there is limited research specifically investigating the correlation between these IR indices and cardiovascular outcomes in patients undergoing PCI with DES, and no studies have compared their predictive abilities for MACCEs in this patient population. Therefore, there is an urgent need to explore the association between these IR indices and cardiovascular events in individuals undergoing PCI with DES, and to assess whether incorporating these IR indices into the basic predictive model improves its performance.

## Methods

### Study population

**Fig. 1:**
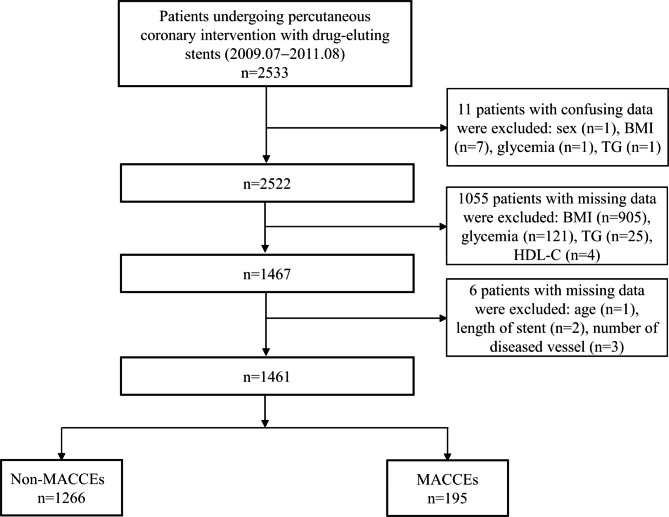
The flowchart of study participants

The data used in the study were obtained from a public dataset (10.5061/dryad.13d31) uploaded by Yao HM et al. [26, 27]. This study with a waiver of informed consent has obtained the approval from the ethics committee of the First Affiliated Hospital of Zhengzhou University. Considering the nature of public dataset, no further research ethic was needed in the present study. The detailed study design has been described by Yao HM et al. [26]. In brief, a total of 2533 consecutive participants with CAD who underwent PCI with DES were recruited between July 2009 and August 2011. The participants were followed up for a median of 29.8 months (25.6−34.0 months). PCI procedures were performed by the experienced surgeons following standard protocols. Loading doses of aspirin (300 mg) and clopidogrel (300 mg) were administered before the PCI, unless the patients were already on standard antiplatelet medication. Medication use after discharge was in accordance with the guidelines at the time. As shown in Fig. 1, a total of 1461 patients were enrolled in the present study after excluded the confusing or missing data.

### Data collection and definitions

The data on demographics, medical history and clinical presentation were recorded at admission. Fasting peripheral venous-blood samples were collected before PCI to obtain the laboratory data, including glycemia, total cholesterol (TC), low-density lipoprotein cholesterol (LDL-C), creatinine, uric acid, triglyceride (TG) and high-density lipoprotein cholesterol (HDL-C). Angiographic and procedural information, such as the location of the culprit vessel, characteristics of lesions, length and diameters of stents, were extracted from the medical records. Information on medication was also collected. The standardized spreadsheets were designed to collect these retrospective data.

Hypertension was diagnosed based on a self-reported physician diagnosis, recent use of an antihypertensive agent, or a BP ≥ 140/90 mmHg [28]. DM was defined as glycemia > 6.1 mmol/L, HbA1c level ≥ 6.5%, or recent use of a hypoglycemic agent (insulin and/or any hypoglycemic drug) [26]. The elderly patients referred to individuals with age ≥ 60 years [29]. BMI was calculated as: BMI (kg/m^2^) = weight/height^2^. Smokers were considered as those who had smoked within the past 10 years. Repeat revascularization in the target vessel was adjudicated as target vessel revascularization (TVR). The formulars of the four indices [13, 14, 16, 17] are summarized as Supplementary file 1, Table S1.

### Endpoints and follow up

The endpoint of the study was defined as MACCEs, including all-cause death, AMI, stroke and TVR. The follow-up data were obtained through outpatient clinic visits, telephone interviews, or readmission, and the endpoints were adjudicated by an independent committee.

### Statistical analysis

Data analyses were performed using STATA MP version 17.0, RStudio 4.2.1 and SPSS 24.0. The normal distribution and equality of variance of continuous datasets were evaluated using the Kolmogorov-Smirnov test and Levene test, respectively. The continuous datasets were described using mean ± standard deviation (SD) or median (25th and 75th percentiles), and compared by one-way analysis of variance or Mann-Whitney U test, as appropriate. Nominal variables were described as counts and percentages, and the chi-square test or Fisher’s exact test was used to identify differences between the groups, as appropriate.

The four IR indices were standardized (Z-score) and added to the unadjusted or adjusted models to evaluate the impact of per 1.0-SD increment in the indices on MACCEs. The univariate logistic regression was conducted prior to the multivariate model to select the covariates. Based on a significance level of *P* < 0.05, the following covariates were adjusted for: age, hypertension, diabetes mellitus, heart failure, previous AMI, creatinine, uric acid (UA), angiotensin converting enzyme inhibitor (ACEI), number of diseased vessels, left anterior descending (LAD), right coronary artery (RCA), length and diameters of stents to identify the association between the four IR indexes and MACCEs in overall population. All the adjusted variables were evaluated for collinearity, and no clear evidence of multicollinearity was found in overall population (all-variance inflation factor of the included variables was < 5) (Supplementary file 2, Figure S1A).

Next, we also conducted subgroup analyses stratified by age, sex, DM and hypertension, and the *P* value for interaction was calculated. Then the association between the four IR indices and MACCEs was further explored in elderly and female patients, respectively, and no multicollinearity was detected using the above criteria (Supplementary file 2, Figure S1B and C). Multivariate adjusted restricted cubic splines (RCS) were used to assess whether there was a linear or nonlinear correlation between METS-IR and MACCEs in the gender and age subgroups (with a threshold of *P* < 0.10). The receiver operating characteristic (ROC) curves were used for diagnostic value analysis, and the area under the curve, as measured by the C-statistic, was computed to quantify the predictive power of logistic models for MACCEs [30]. Additionally, the net reclassification improvement (NRI) and integrated discrimination improvement (IDI) index were calculated to further assess the additional predictive value of the four indices beyond established risk factors for MACCEs. A two-side *P* < 0.05 was considered as statistically significant.

## Results

The study excluded 11 patients with confusing data and an additional 1061 patients with missing data, as shown in Fig. 1. The clinical features of the included and excluded patients were presented at Supplementary file 1, Table S2. The included patients had a higher prevalence of DM, a higher proportion of smoking, and use of ACEI and stain, a higher prevalence of acute coronary syndromes (ACS), and higher levels of lipid parameters. They also had a lower incidence of MACCEs, a lower prevalence of stable angina (SA), previous AMI and stroke. No significant differences in age, BMI, proportion of females, and angiographic characteristics were observed between the excluded and included patients.

### Baseline characteristics of the included participants by MACCEs

The study included a total of 1461 patients receiving at least one DES at baseline. During the 34-month follow-up period, 195 cases (13.35%) experienced incident MACCEs. The baseline characteristics of the participants were compared between those who experienced MACCEs and those who did not, and the results are presented in Table 1. As expected, several variables showed obvious differences between the patients with MACCEs and those without MACCEs. In brief, compared patients without MACCEs, those with MACCEs tended to be older and had a higher prevalence of previous AMI, heart failure, hypertension, and DM. They also had higher levels of creatinine and UA, and had a higher proportion of ACEI use. Additionally, there was a higher prevalence of chronic total occlusions (CTO) and ≥ 3-vessel disease, as well as a higher occurrence of target lesions in LAD and RCA. Furthermore, the length of stents used in patients with MACCEs was longer compared to those without MACCEs (all *P* < 0.05). However, no discernible differences were found in the proportion of females, BMI, lipid parameters, glycemia, and any of the four IR indices between the MACCEs and non-MACCEs groups (all *P* > 0.05). In addition, Supplementary file 1, Table S3 and S4 showed the baseline data for the participants, classified according to age and sex.

**Table 1:** Baseline characteristics of participants by MACCEs.

Characteristics	Overall (n = 1461)	Non-MACCEs (n = 1266)	MACCEs (n = 195)	*P* value
Demographics				
Age, years	60.10 ± 11.11	59.40 ± 10.94	64.67 ± 11.14	< 0.001
Female, (%)	459 (31.4)	395 (31.2)	64 (32.8)	0.650
BMI, kg/m^2^	23.85 ± 3.80	23.79 ± 3.79	24.27 ± 3.89	0.102
Medical history				
Heart failure, n (%)	161 (11.0)	129 (10.2)	32 (16.4)	0.010
Atrial fibrillation, n (%)	25 (1.7)	21 (1.7)	4 (2.1)	0.764
Previous AMI, n (%)	130 (8.9)	103 (8.1)	27 (13.8)	0.009
Previous stroke, n (%)	64 (4.4)	55 (4.3)	9 (4.6)	0.863
Previous PCI, n (%)	82 (5.6)	68 (5.4)	14 (7.2)	0.308
Hypertension, n (%)	726 (49.7)	616 (48.7)	110 (56.4)	0.045
Diabetes mellitus, n (%)	329 (22.5)	268 (21.2)	61 (31.4)	0.001
Smoking, n (%)	502 (34.4)	430 (34.0)	72 (36.9)	0.418
Clinical presentation				0.842
STEMI, n (%)	377 (25.8)	329 (26.0)	48 (24.6)	
NSTE-ACS, n (%)	909 (62.2)	784 (61.9)	125 (64.1)	
SA, n (%)	175 (12.0)	153 (12.1)	22 (11.3)	
Laboratory data				
Glycemia, mmol/L	6.10 ± 2.76	6.10 ± 2.80	6.11 ± 2.47	0.963
Creatinine, µmol/L	72.36 ± 30.25	71.43 ± 28.42	78.51 ± 39.85	0.003
Uric acid, µmol/L	305.60 ± 97.38	303.33 ± 91.92	320.46 ± 126.79	0.023
TG, mmol/L	1.61 (1.16, 2.33)	1.62 (1.17, 2.35)	1.56 (1.09, 2.25)	0.363
TC, mmol/L	4.30 ± 1.08	4.29 ± 1.06	4.39 ± 1.20	0.232
HDL-C, mmol/L	1.08 ± 0.34	1.08 ± 0.32	1.09 ± 0.42	0.610
LDL-C, mmol/L	2.74 ± 0.95	2.72 ± 0.94	2.84 ± 1.03	0.091
Treatment				
Aspirin, n (%)	1438 (98.6)	1243 (98.3)	195 (100.0)	0.070
Clopidogrel, n (%)	1412 (96.6)	1224 (96.7)	188 (96.4)	0.774
Beta blocker, n (%)	1017 (69.6)	872 (68.9)	145 (74.4)	0.121
ACEI, n (%)	854 (58.5)	714 (56.4)	140 (71.8)	< 0.001
CCB, n (%)	360 (24.6)	310 (24.5)	50 (25.6)	0.728
Statin, n (%)	1365 (93.4)	1177 (93.0)	188 (96.4)	0.071
Number of diseased vessels				< 0.001
1-vessel disease, n (%)	545 (37.3)	496 (39.2)	49 (25.1)	
2-vessel disease, n (%)	554 (37.9)	484 (38.2)	70 (35.9)	
3-vessel disease, n (%)	362 (24.8)	286 (22.6)	76 (39.0)	
Location of target lesions				
LM, n (%)	46 (3.1)	36 (2.8)	10 (5.1)	0.089
LAD, n (%)	1221 (83.6)	1046 (82.6)	175 (89.7)	0.012
LCX, n (%)	717 (49.1)	609 (48.1)	108 (55.4)	0.058
RCA, n (%)	736 (50.4)	614 (48.5)	122 (62.6)	< 0.001
Characteristics of lesions				
Occlusion, n (%)	197 (13.5)	173 (13.7)	24 (12.3)	0.605
CTO, n (%)	121 (8.3)	88 (7.0)	33 (16.9)	< 0.001
Ostial lesion, n (%)	170 (11.6)	146 (11.5)	24 (12.3)	0.753
Bifurcation lesion, n (%)	251 (17.2)	217 (17.1)	34 (17.4)	0.919
Number of treated vessels				< 0.001
1-vessel disease, n (%)	853 (58.4)	747 (59.0)	106 (54.4)	
2-vessel disease, n (%)	481 (32.9)	424 (33.5)	57 (29.2)	
≥ 3-vessel disease, n (%)	127 (8.7)	95 (7.5)	32 (16.4)	
Length of stents, (mm)	48.55 ± 31.24	47.11 ± 29.95	57.90 ± 37.33	< 0.001
Diameter of stents, (mm)	3.12 ± 1.09	3.14 ± 1.16	3.00 ± 0.40	0.098
TyG index	8.94 ± 0.67	8.95 ± 0.66	8.94 ± 0.69	0.912
TyG-BMI index	213.54 ± 38.93	212.95 ± 38.51	217.40 ± 41.50	0.137
TG/HDL-C ratio	3.65 (2.37, 5.67)	3.70 (2.37, 5.67)	3.18 (2.38, 5.67)	0.280
METS-IR	38.53 ± 7.73	38.42 ± 7.69	39.25 ± 8.01	0.162

ACEI, angiotensin converting enzyme inhibitor; AMI, acute myocardial infraction; BMI, body mass index; CCB, calcium channel blocker; CTO, chronic total occlusions; HDL-C, high-density lipoprotein cholesterol; LAD, left anterior descending; LCX, left circumflex artery; LDL-C, low-density lipoprotein cholesterol; LM, left main coronary artery; MACCEs, major adverse cardiac and cerebrovascular events; METS-IR, metabolic score for insulin resistance; NSTEMI, ono-ST elevation myocardial infarction; PCI, percutaneous coronary intervention; RCA, right coronary artery; SA, stable angina; STEMI, ST elevation myocardial infarction; TC, total cholesterol; TG, triglyceride; TG/HDL-C, triglyceride to high-density lipoprotein cholesterol ratio; TyG, triglyceride and glucose; TyG-BMI, triglyceride glucose-body mass index

### Association of IR indices with MACCES in overall population

The logistic analyses were conducted to assess the impact of per 1.0-SD increment in the four IR indices on MACCEs, as shown in Table 2. To screen for the adjusted covariates, the univariate logistic regression analysis was performed (Supplementary file 1, Table S5), and the identified risk factors included age, heart failure, previous AMI, hypertension, DM, creatinine, UA, use of ACEI, target lesions in LAD and RCA, CTO, number of diseased vessels, and the diameters and length of stents. Regrettably, no great association were found between the four IR indices and MACCEs in either univariate or multivariate logistic models.

**Table 2:** Association between insulin resistance indexes and MACCEs in overall population

	OR (95% CI)^a^	*P* value	OR (95% CI)^b^	*P* value	OR (95% CI)^c^	*P* value
TyG index	0.99 (0.85–1.15)	0.912	0.98 (0.83–1.16)	0.844	0.94 (0.79–1.12)	0.508
TyG-BMI index	1.12 (0.97–1.30)	0.137	1.05 (0.89–1.23)	0.575	1.03 (0.87–1.22)	0.738
TG/HDL-C ratio	1.04 (0.90–1.19)	0.610	1.09 (0.95–1.25)	0.225	1.05 (0.90–1.21)	0.550
METS-IR	1.11 (0.96–1.29)	0.162	1.08 (0.92–1.27)	0.349	1.05 (0.89–1.24)	0.578

CI, confidence interval; OR, odds ratio

^a^ Model 1: unadjusted

^b^ Model 2: adjusted for age, hypertension, and diabetes mellitus

^c^ Model 3: model 2 + further adjusted for heart failure, previous AMI, creatinine, uric acid, ACEI, number of diseased vessels, LAD, RCA, length of stents, and diameters of stents

### Subgroup analyses

**Fig. 2:**
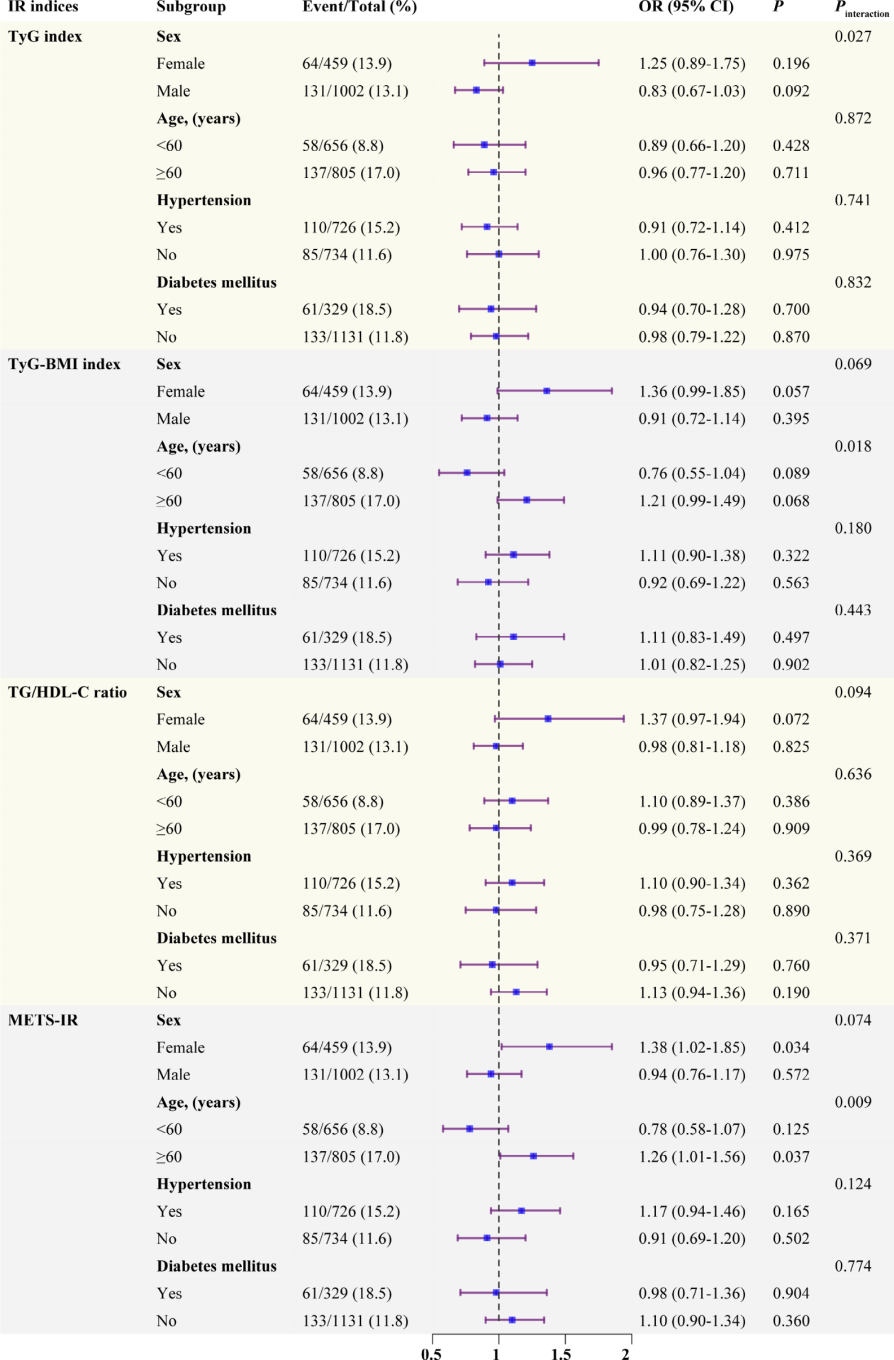
Association between IR indices and MACCEs among people undergoing PCI in different subgroups. Each subgroup was adjusted for age, hypertension, diabetes mellitus, heart failure, previous AMI, creatinine, uric acid, ACEI, number of diseased vessels, LAD, RCA, length of stents, and diameters of stents. Odds ratios are presented as per 1.0-SD increase in the IR indices for MACCEs. ACEI, angiotensin converting enzyme inhibitor; AMI, acute myocardial infraction; CI, confidence interval; IR, insulin resistance; LAD, left anterior descending; MACCEs, major adverse cardiac and cerebrovascular events; METS-IR, metabolic score for insulin resistance; RCA, right coronary artery; TG/HDL-C, triglyceride to high-density lipoprotein cholesterol ratio; TyG, triglyceride and glucose; TyG-BMI, triglyceride glucose-body mass index

**Fig. 3:**
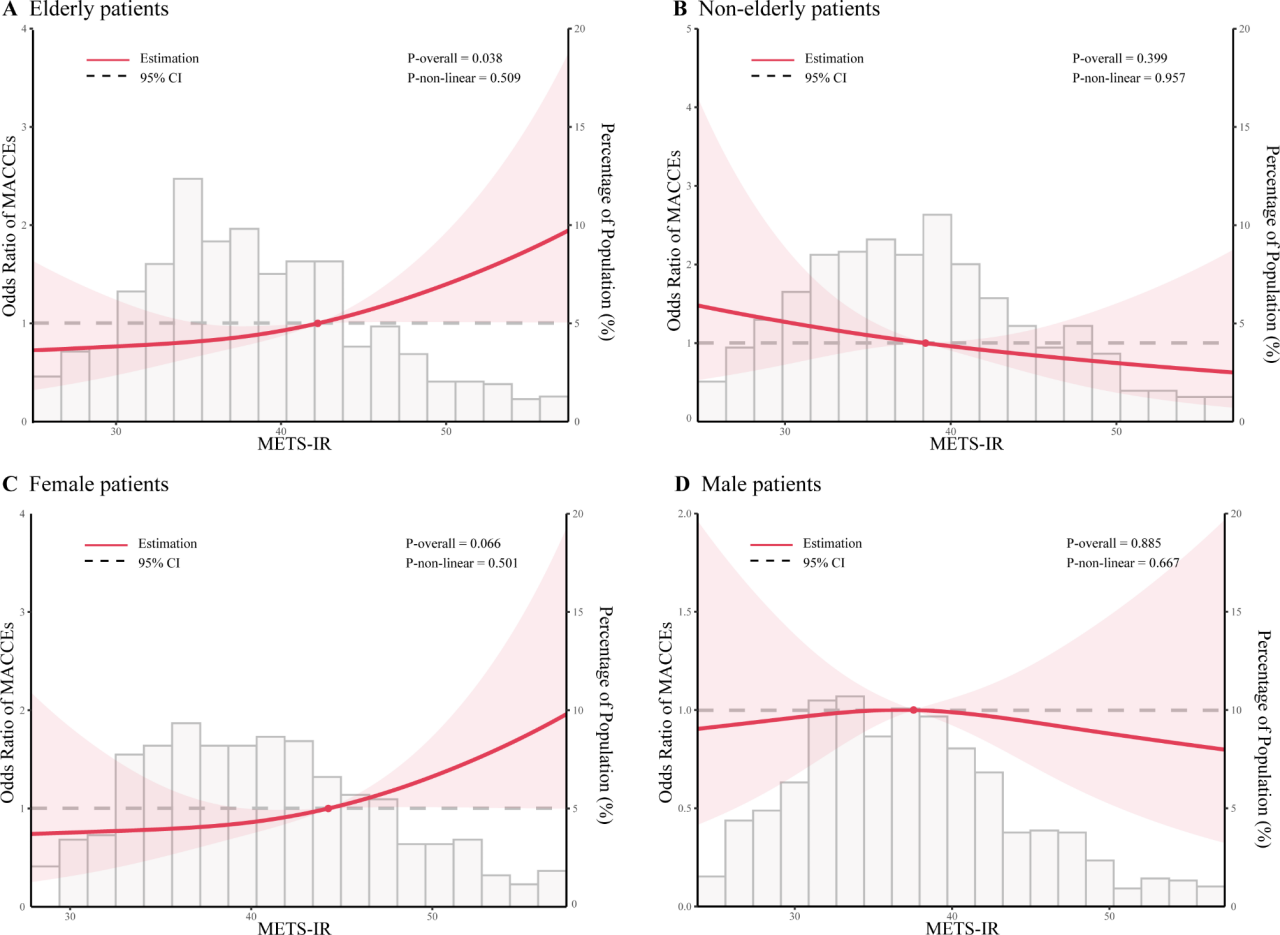
Restricted cubic spline curves for MACCEs by METS-IR after covariate adjustment. **(A)** Relationship in the elderly patients; **(B)** Relationship in the non-elderly patients; **(C)** Relationship in the female patients; **(D)** Relationship in the male patients. The threshold of statistical significance was set as *P* < 0.10. In **A** and **B**, age, heart failure, previous AMI, creatinine, uric acid, ACEI, number of diseased vessels, LAD, RCA, CTO, and length of stents were adjusted; in **C** and **D**, age, previous AMI, creatinine, uric acid, ACEI, number of diseased vessels, RCA, CTO, and length of stents were adjusted. Odds ratios are indicated by solid lines and 95% CIs by shaded areas. ACEI, angiotensin converting enzyme inhibitor; AMI, acute myocardial infraction; CI, confidence interval; CTO, chronic total occlusions; IR, insulin resistance; LAD, left anterior descending; MACCEs, major adverse cardiac and cerebrovascular events; METS-IR, metabolic score for insulin resistance; RCA, right coronary artery

Next, we conducted the exploratory subgroups analyses stratified by age, sex, DM and hypertension. As shown in Fig. 2, the results indicated a significant interaction between age subgroups and the impact of TyG index on the incidence of MACCEs (*P* for interaction = 0.027). Similarly, significant interactions were detected between sex subgroups and either TyG-BMI index or METS-IR (*P* for interaction = 0.018 and 0.009, respectively). Furthermore, a positive correlation was found between per 1.0-SD increment in METS-IR and the incidence of MACCEs in elderly (OR: 1.26, 95% CI: 1.01−1.56, *P* = 0.037) and female patients (OR: 1.38, 95% CI: 1.02−1.85, *P* = 0.034), respectively. Encouraged by the results, we assessed whether a linear or nonlinear association existed between METS-IR and the subgroups stratified by age and sex using multivariate adjusted RCS. The results, as shown in Fig. 3, indicated a significantly linear relationship between METS-IR and MACCEs in elderly patients (*P* for overall = 0.038, *P* for nonlinear = 0.509) and female patients (*P* for overall = 0.066, *P* for nonlinear = 0.501). However, no apparent association was observed in non-elderly and male patients (all *P* for overall > 0.10).

### Association of IR indices with MACCEs in elderly patients

Then the correlations between the four IR indices and MACCEs in elderly patients were explored. Covariates were selected based on the results from univariate logistic model (Supplementary file 1, Table S6). Three models were established, including an unadjusted, partly adjusted and fully adjusted model (Table 3). The results indicated that per 1.0-SD increment in TyG-BMI index and METS-IR showed a significant association with MACCEs in elderly patients. The OR for the TyG-BMI index was 1.24 (95% CI: 1.02−1.50, *P* = 0.033), while for METS-IR, the OR was 1.27 (95% CI: 1.04−1.56, *P* = 0.020).

**Table 3:** Association between insulin resistance indexes and MACCEs in elderly patients

	OR (95% CI)^a^	*P* value	OR (95% CI)^b^	*P* value	OR (95% CI)^c^	*P* value
TyG index	1.01 (0.84–1.22)	0.904	1.09 (0.90–1.32)	0.390	1.04 (0.85–1.27)	0.719
TyG-BMI index	1.21 (1.01–1.44)	0.036	1.26 (1.06–1.51)	0.011	1.24 (1.02–1.50)	0.033
TG/HDL-C ratio	1.00 (0.83–1.22)	0.967	1.06 (0.87–1.28)	0.570	1.01 (0.81–1.26)	0.918
METS-IR	1.25 (1.04–1.50)	0.017	1.32 (1.09–1.59)	0.004	1.27 (1.04–1.56)	0.020

CI, confidence interval; OR, odds ratio

^a^ Model 4: unadjusted

^b^ Model 5: adjusted for age, heart failure, and previous AMI

^c^ Model 6: model 5 + further adjusted for creatinine, uric acid, ACEI, number of diseased vessels, LAD, RCA, CTO, and length of stents

### Association of IR indices with MACCEs in female patients

We further explored the relationship of these IR indices with MACCEs in female patients. Similar with the above analysis, the univariate logistic model was performed prior to the three models constructed (Supplementary file 1, Table S7). All the four IR indices had a positive correlation with MACCEs in the female patients in whichever of the three models (Table 4).

**Table 4:** Association between insulin resistance indexes and MACCEs in female patients

	OR (95% CI)^a^	*P* value	OR (95% CI)^b^	*P* value	OR (95% CI)^c^	*P* value
TyG index	1.36 (1.05–1.76)	0.021	1.39 (1.06–1.83)	0.018	1.39 (1.04–1.86)	0.024
TyG-BMI index	1.37 (1.07–1.76)	0.013	1.35 (1.04–1.75)	0.023	1.37 (1.04–1.80)	0.027
TG/HDL-C ratio	1.40 (1.05–1.85)	0.021	1.52 (1.12–2.07)	0.008	1.49 (1.08–2.06)	0.015
METS-IR	1.32 (1.04–1.69)	0.024	1.35 (1.05–1.74)	0.020	1.38 (1.05–1.81)	0.021

CI, confidence interval; OR, odds ratio

^a^ Model 7: unadjusted

^b^ Model 8: adjusted for age, previous AMI

^c^ Model 9: model 8 + further adjusted for creatinine, uric acid, ACEI, number of diseased vessels, RCA, CTO, and length of stents

### Incremental predictive performance of IR indexes in the risk assessment of MACCEs

**Fig. 4:**
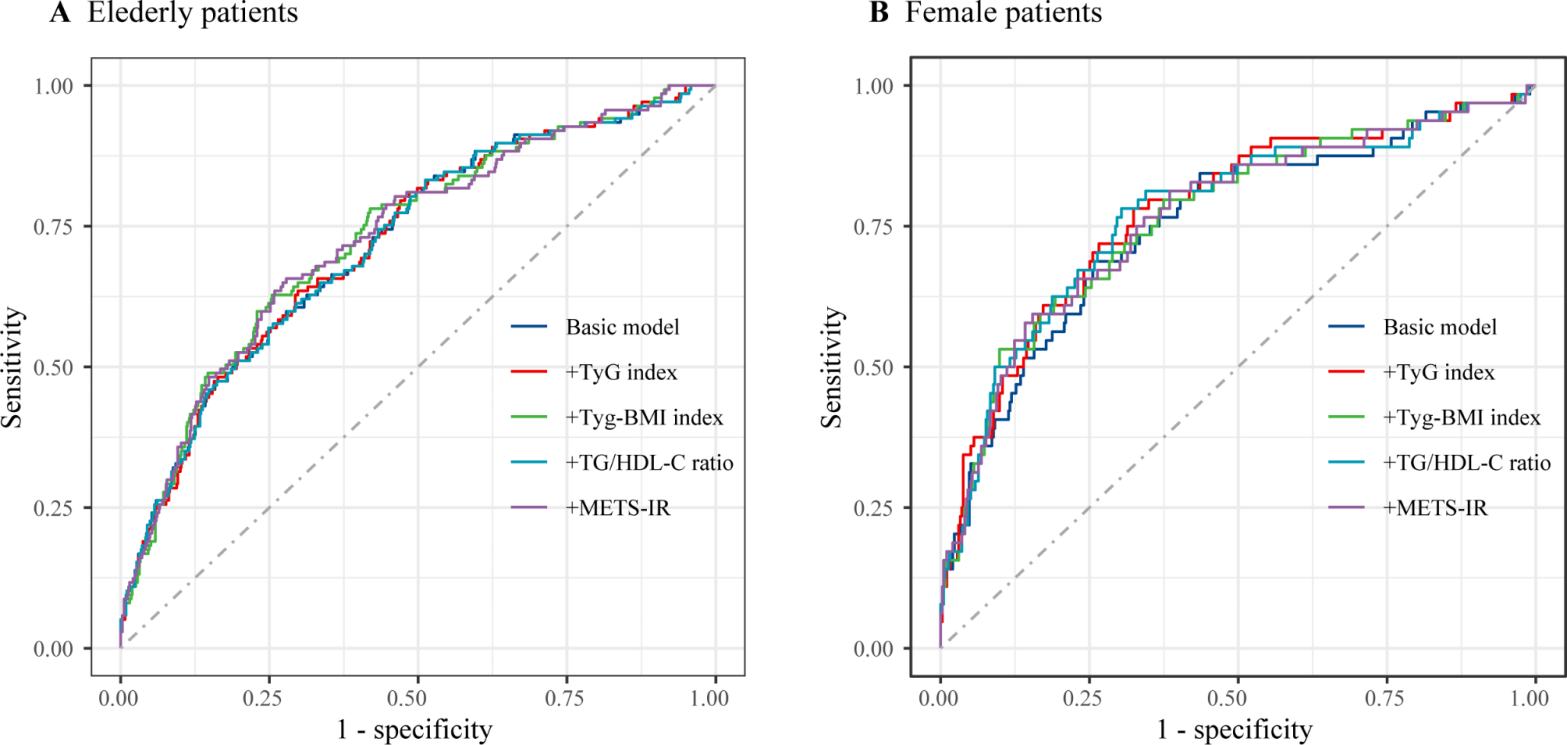
The receiver operating characteristic curves of the IR indices as a marker to predict MACCEs. **(A)** Basic risk model vs. + the IR indices in the elderly patients. Basic risk model includes age, heart failure, previous AMI, creatinine, uric acid, ACEI, number of diseased vessels, LAD, RCA, CTO, and length of stents; **(B)** Basic risk model vs. + the IR indices in the female patients. Basic risk model includes age, previous AMI, creatinine, uric acid, ACEI, number of diseased vessels, RCA, CTO, and length of stents. ACEI, angiotensin converting enzyme inhibitor; AMI, acute myocardial infraction; CI, confidence interval; CTO, chronic total occlusions; IR, insulin resistance; LAD, left anterior descending; MACCEs, major adverse cardiac and cerebrovascular events; METS-IR, metabolic score for insulin resistance; RCA, right coronary artery

In the analysis of elderly patients, the ROC curves were constructed to assess the predictive power of the basic model (including age, heart failure, previous AMI, creatinine, UA, use of ACEI, number of diseased vessels, LAD, RCA, CTO, and length of stents) and the basic model plus each of the four IR indices for MACCEs, respectively (Fig. 4A). The C-statistic, NRI and IDI were presented in Table 5. Unfortunately, the results showed no significant incremental predictive ability of the four IR indices to the basic risk model in elderly patients. The similarly results also were observed in female patients (Fig. 4B; Table 6).

**Table 5:** Improvement in discrimination and risk reclassification for MACCEs after adding IR indices in elderly patients

Model	C-statistic (95% CI)	*P* value	NRI (95% CI)	*P* value	IDI (95% CI)	*P* value
Basic model	0.719 (0.672–0.767)	Ref.	Ref.		Ref.	
+TyG index	0.720 (0.673–0.768)	0.562	0.065 (-0.092–0.244)	0.452	0.000 (-0.001–0.001)	0.905
+TyG-BMI index	0.730 (0.683–0.777)	0.150	0.243 (0.009–0.419)	0.020	0.006 (-0.001–0.014)	0.109
+TG/HDL-C ratio	0.720 (0.672–0.767)	0.854	-0.062 (-0.147–0.288)	0.578	0.000 (-0.001–0.001)	0.855
+METS-IR	0.729 (0.681–0.779)	0.228	0.158 (-0.008–0.373)	0.109	0.009 (0.001–0.016)	0.034

CI, confidence interval; IDI, integrated discrimination improvement; NRI, net reclassification improvement

The basic model included age, heart failure, previous AMI, creatinine, uric acid, ACEI, number of diseased vessels, LAD, RCA, CTO, and length of stents

**Table 6:** Improvement in discrimination and risk reclassification for MACCEs after adding IR indices in female patients

Model	*C*-statistic (95% CI)	*P* value	NRI (95% CI)	*P* value	IDI (95% CI)	*P* value
Basic model	0.753 (0.684–0.823)	Ref.	Ref.		Ref.	
+TyG index	0.776 (0.709–0.842)	0.040	0.178 (-0.046–0.569)	0.257	0.013 (-0.002–0.027)	0.087
+TyG-BMI index	0.767 (0.698–0.835)	0.169	0.241 (-0.027–0.538)	0.092	0.012 (-0.002–0.027)	0.103
+TG/HDL-C ratio	0.772 (0.704–0.841)	0.175	0.101 (-0.182–0.507)	0.564	0.014 (-0.004–0.033)	0.122
+METS-IR	0.766 (0.698–0.835)	0.194	0.174 (-0.055–0.523)	0.235	0.014 (-0.001–0.028)	0.066

CI, confidence interval; IDI, integrated discrimination improvement; NRI, net reclassification improvement

The basic model included age, previous AMI, creatinine, uric acid, ACEI, number of diseased vessels, RCA, CTO, and length of stents

## Discussion

Considering the limited availability of data on evaluating the predictive performance of IR indices in patients undergoing PCI, we designed the present study that assessed the association of the four IR indices with MACCEs in patients undergoing PCI with at least one DES. This study not only provides new evidence for risk stratification of these patients but also represents the first investigation of the association between METS-IR and MACCEs in PCI patients. By addressing this research gap, our findings may exert far-reaching significance for the secondary prevention of CVD. The main findings could be summarized as follows: (1) the TyG-BMI index and METS-IR were markedly associated with MACCEs in elderly patients, while all the four IR indexes were obviously associated with MACCEs in female patients; (2) more importantly, the METS-IR demonstrated a strong linear association with MACCEs in either elderly or female individuals, suggesting that METS-IR might be a promising biomarker in predicting the adverse cardiovascular outcomes in those patients; (3) the four IR indices did not significantly optimize the predictive performance of basic risk model for MACCEs in either elderly or female patients.

### The value and need of IR assessment on cardiovascular outcomes

Increasing evidence suggested that IR, as the major characteristic of type 2 DM, has a major role in the pathogenesis of CVD [31]. Currently, growing evidence also suggests that elevated IR can initiate and contribute to the development of CVD, as well as predict cardiovascular events in patients with pre-existing CVD [32–34]. More importantly, although remarkable advancements have been made in clinical practice, such as timely PCI and medication treatment, the incidence of CVD in general population continues to rise, and the long-term cardiovascular events, like restenosis in stents, heart failure, stroke and cardiac death, remain high. Therefore, there is an urgent need to identify the IR promptly and accurately, with an expectation of improving primary and secondary prevention through establishing effective cardiovascular risk stratification. While the hyperinsulinemic-euglycemic clamp is regarded as the gold-standard method of assessing IR [12], its complexity limits its application in clinical practice and epidemiological investigation. Consequently, several reliable and valid alternatives for non-insulin-based IR assessment have been developed [13–17]. However, to date, the relationship between IR and cardiovascular outcomes of patients undergoing PCI have not been well elucidated. Thus, this study was designed to explore the association of METS-IR with MACCEs and compare the predictive abilities of the four IR indices for MACCEs in elderly or female patients undergoing PCI.

### The previous evidence on association of IR with MACCEs

Data from a study of 1092 ACS patients undergoing PCI suggested that patients with the upper quartile of TyG index had an increased risk of MACCEs within 1 year after PCI (hazard ratio (HR): 1.53, 95% CI: 1.00−2.06, *P* = 0.003) [9]. Another study by Xiong et al., which included 633 consecutive patients with type 2 DM undergoing PCI and followed up for 18.83-month, found that TyG index independently predicted the major adverse cardiac events (HR: 1.80, 95% CI: 1.26−2.57, *P* = 0.001). Furthermore, the inclusion of TyG index in basic risk model significantly improved the predictive performance [35]. Evidence from the Korean population indicated that TyG-BMI index was superior to TyG index for IR prediction [36]. The TyG-BMI index has also demonstrated a robust and independent association with ischemic stroke [37]. However, the inconsistent results were found in a rural Chinese cohort study involving 14,595 participants. The study suggested that the relative risk (95% CI) for stroke with per 1.0-SD increment in TyG index was 1.23 (1.15−1.32) and in TyG-BMI index was 1.17 (1.09−1.26) (both *P* < 0.05), and the C-statistics (95% CIs) for predicting stroke were 0.614 (0.606−0.621) for TyG index and 0.598 (0.590−0.606) for the TyG-BMI index, respectively [25]. Furthermore, a study by Yunke et al. involving 317 participants revealed that TG/HDL-C ratio could independently predict cardiovascular events [38]. Two other studies also demonstrated that the TG/HDL-C ratio was an effective biomarker for evaluating the severity of CAD [22, 39]. However, there are inconsistent findings regarding the priority between TyG index and TG/HDL-C ratio.

### Stronger associations of METS-IR with MACCEs than other IR indices

More recently, the METS-IR has been proposed as a more promising and reliable indicator for evaluating IR and predicting cardiovascular outcomes [13]. Mounting evidence suggests that METS-IR is positively associated with the severity of CAD and has a better predictive ability than the other three indices [22, 39]. These findings are consistent with our own findings, as only METS-IR showed a positive correlation with MACCEs in both female and elderly patients in the subgroup analyses (Fig. 2). The positive association of METS-IR with MACCEs remains robust in elderly and female patients (Tables 4 and 5), but not in the overall population. There are several possible reasons for the findings. Firstly, age is a well-known risk factor for CAD, and elderly individuals with CAD are more likely to experience cardiovascular events [40]. Secondly, the elderly patients in our study had a higher proportion of multi-vessel (2- and 3-vessel) disease, higher BMI, higher prevalence of DM, and longer length of stents. Regarding the sex-specific difference, female patients in our study were much older than male patients (63.24 ± 10.04 vs. 58.67 ± 11.28). Thirdly, the prospective effect of estrogen on circulating and endocrine systems may diminish or disappear for females in menopausal transition or postmenopausal period [41, 42]. Aging women, especially those in the menopausal transition or postmenopausal period, are more likely to have increased abdominal fat accumulation and a higher prevalence of metabolic dysfunction, such as dyslipidemia, IR, and chronic low-grade inflammation [41, 43]. Several previous studies have also reported a more pronounced association of IR with cardiovascular events in females compared to males, which is consistent with our results [34, 44]. Moreover, differences in endothelial function between elderly and younger patients may contribute to the observed results. Age-related arterial stiffness and endothelial dysfunction can impact treatment response and long-term outcomes in elderly patients [45]. Additionally, chronic low-grade inflammation is common in elderly or perimenopausal women, which may increase the risk of MACCEs [43]. Finally, elderly and female CAD patients often have a higher burden of comorbidities and coexisting conditions, such as hypertension, DM, abdominal obesity, and chronic kidney disease [46], which can interact with CAD and influence treatment outcomes.

Furthermore, a prospective cohort study of 6489 adults suggested that the highest quintile and per 1.0-SD increasement in METS-IR were highly correlated with a higher risk of CVD incidence (HR: 1.80, 95% CI: 1.24−2.61, *P* < 0.01; HR: 1.17, 95% CI: 1.05−1.31, *P* < 0.01, respectively) [47]. These inconsistent results may be attributed to the heterogeneity of the included population and study design. Therefore, there is an urgent need to conduct our study to provide additional evidence on whether non-insulin IR indices can predict cardiovascular outcomes in patients undergoing PCI.

### General mechanisms of IR on MACCEs

The association between these indices and MACCEs in patients undergoing PCI with at least one DES may be explained, at least in part, by the following mechanisms. First, involvement of IR in the pathogenesis of CAD: high circulating insulin concentrations can reduce the production of nitric oxide *via* the activation of serum and glucocorticoid kinase 1, leading to decreased nitric oxide concentration. This, in turn, can result in matrix protein deposition and fibrosis [48]. Second, the metabolic impairment of lipid and glucose induced by IR: IR can lead to the overproduction of reactive oxide species through the activation of signaling pathways, such as the protein kinase C pathway and the nuclear factor (NF)­κB pathway. These pathways can trigger cardiovascular events [49]. Third, the ectopic synthesis of angiotensinogen and the inappropriate activation of the RAAS caused by IR contribute to fluid retention and high blood pressure [10, 11]. Finally, impact of insulin on thrombosis and platelet aggregation: insulin can impair fibrinolysis by increasing the circulating concentration of plasminogen activator inhibitor 1. This can lead to a pro-thrombotic state and promote platelet aggregation in the cardiovascular system [50].

### Strengths and limitations

In this study, we explored the correlation of METS-IR with MACCEs and respectively assessed the predictive ability of the four IR indices for MACCEs in CAD patients undergoing PCI. While we found no positive association between these IR indices and MACCEs in overall population, and no additional predictive performance was observed, our results suggest that both METS-IR and TyG-BMI index could serve as effective and reliable predictors for MACCEs in elderly patients (age ≥ 60 years) and female patients with CAD undergoing PCI. These findings have far-reaching significance for secondary prevention and risk stratification in these patient populations. However, there are several limitations that should be acknowledged. Firstly, the lack of follow-up time in the dataset prevented us from conducting the time-to-event analysis, which may underestimate the association between IR and MACCEs. Secondly, due to the observational nature of the study, obtain dynamic data on the four IR indices during the follow-up period were unavailable. Having longitudinal data could have added value to the risk stratification of MACCEs. Therefore, future prospective, large-scale, multicenter randomized controlled trials are needed to address this limitation. Thirdly, the dataset did not provide information on hypoglycemic treatment, which could potentially introduce bias in the analysis. Additionally, the lack of data on patients’ dietary habits is another notable limitation, as diet could be a crucial confounder. Including detailed dietary assessments in future studies would be helpful to a more comprehensive understanding of the association between IR and MACCEs and enhance the validity of the results. Finally, despite adjusting for many potential confounders, there may still be residual or unmeasured confounders that could influence the observed associations, such as sleep duration and physical activities. Future studies should consider incorporating these factors to further improve the accuracy and validity of the results.

## Conclusions

As widely recognized surrogates of IR, all the four indices demonstrated a clear association with MACCEs in female individuals, while only the TyG-BMI index and METS-IR exhibited such an association in elderly patients. Although incorporating these indices into the basic risk model did not lead to an improvement in predictive performance for MACCEs in either female or elderly patients, METS-IR appears to hold the most promise as an index for secondary prevention and risk stratification of MACCEs in these patient populations. Further investigations are urgently required to validate and build upon these findings.

## Electronic supplementary material

Below is the link to the electronic supplementary material.

Supplementary Material 1

Supplementary Material 2

## Data Availability

The datasets generated and/or analyzed during the current study are available in the Dryad repository 10.5061/dryad.13d31.

## List of abbreviations

ACEI: Angiotensin converting enzyme inhibitor
ACS: Acute coronary syndrome
AMI: Acute myocardial infraction
BMI: Body mass index
CAD: Coronary artery disease
CTO: Chronic total occlusions
CVD: Cardiovascular disease
CI: Confidence interval
DBP: Diastolic blood pressure
DES: Drug-eluting stent
DM: Diabetes mellitus
HDL-C: High-density lipoprotein cholesterol
HR: Hazard ratio
IDI: Integrated discrimination improvement
IR: Insulin resistance
LAD: Left anterior descending
LDL-C: Low-density lipoprotein cholesterol
MACCEs: Major adverse cardiac and cerebrovascular events
METS-IR: Metabolic score for insulin resistance
NRI: Net reclassification improvement
OR: Odds ratio
PCI: Percutaneous coronary intervention
RAAS: Renin-angiotensin-aldosterone system
RCS: Restricted cubic spline
RCA: Right coronary artery
ROC: Receiver operating characteristic
SA: Stable angina
SBP: Systolic blood pressure
TC: Total cholesterol
TG: Triglyceride
TG/HDL-C: Triglyceride to high-density lipoprotein cholesterol ratio
TyG: Triglyceride and glucose
TyG-BMI: Triglyceride glucose-body mass index
TVR: Target vessel revascularization
UA: Uric acid
