# Supplementary material for: Association between non-insulin-based insulin resistance indices and cardiovascular events in patients undergoing percutaneous coronary intervention: a retrospective study
Source: Cardiovasc Diabetol. 2023 Jun 29;22:161. doi: 10.1186/s12933-023-01898-1 (PMC10311786; doi:10.1186/s12933-023-01898-1)
Supplement: Supplementary file 1 — Supplementary Material 1 [file 12933_2023_1898_MOESM1_ESM.docx]

**Additional file Content**

**Supplemental Tables**

**Table S1** Definition of insulin resistance indexes

**Table S2** Characteristics of the included population and excluded population

**Table S3** Baseline characteristics of participants by age

**Table S4** Baseline characteristics of participants by sex

**Table S5** Association between MACCEs and risk factor in in overall population

**Table S6** Association between MACCEs and risk factor in in elderly patients

**Table S7** Association between MACCEs and risk factor in in female patients

**Supplemental Figure**

**Figure S1.** The variance inflation factor (VIF) values for all variables in fully adjusted models. **A** in overall patients; **B** in elderly patients; **C** in female patients

**Abbreviations**: ACEI, angiotensin converting enzyme inhibitor; AMI, acute myocardial infraction; Cr, creatine; CTO, chronic total occlusions; DM, diabetes mellitus; HF, heart failure; LAD, left anterior descending; LDL-C, low-density lipoprotein cholesterol; LM, left main coronary artery; METS-IR, metabolic score for insulin resistance; RCA, right coronary artery; UA, uric acid

**Table S1** Definition of insulin resistance indexes

| **Variables** | **Formulas** |
| --- | --- |
| TyG index | TyG index = Ln (TG × FBG ÷ 2) |
| TyG-BMI index | TyG-BMI index = Ln (TG × FBG ÷ 2) × BMI |
| TG/HDL-C ratio | TG/HDL-C ratio = TG ÷ HDL-C |
| METS-IR | METS-IR = Ln (2 × FPG + TG) × BMI ÷ Ln (HDL-C) |

BMI (kg/m^2^), FBG (mg/dl), TG (mg/dl), and HDL (mg/dl) are used to in the formulas above

BMI, body mass index; HDL-C, high-density lipoprotein cholesterol; TG, triglycerides; TyG, Triglyceride and glucose; TyG-BMI, triglyceride glucose-body mass index; TG/HDH-C, triglyceride to high-density lipoprotein cholesterol ratio; METS-IR, metabolic score for insulin resistance

**Table S2** Characteristics of the included population and excluded population

| **Characteristics** | **Overall**  **(n=2522)** | **Excluded**  **(n=1061)** | **Included**  **(n=1461)** | ***P* value** |
| --- | --- | --- | --- | --- |
| Demographics |  |  |  |  |
| Age, years | 59.97 ± 11.08 | 59.79 ± 11.05 | 60.10 ± 11.11 | 0.487 |
| Female, (%) | 805 (31.9) | 346 (32.6) | 459 (31.4) | 0.525 |
| BMI, kg/m^2^ | 23.89 ± 3.82 | 24.28 ± 3.98 | 23.85 ± 3.80 | 0.180 |
| Medical history |  |  |  |  |
| Heart failure, n (%) | 294 (11.7) | 133 (12.6) | 161 (11.0) | 0.240 |
| Atrial fibrillation, n (%) | 50 (2.0) | 25 (2.4) | 25 (1.7) | 0.251 |
| Previous AMI, n (%) | 234 (9.3) | 104 (9.8) | 130 (8.9) | 0.440 |
| Previous stroke, n (%) | 135 (5.4) | 71 (6.7) | 64 (4.4) | 0.011 |
| Previous PCI, n (%) | 169 (6.7) | 87 (8.2) | 82 (5.6) | 0.010 |
| Hypertension, n (%) | 1244 (49.3) | 518 (48.8) | 726 (49.7) | 0.654 |
| Diabetes mellitus, n (%) | 521 (20.7) | 192 (18.1) | 329 (22.5) | 0.007 |
| Smoking, n (%) | 812 (32.2) | 310 (29.2) | 502 (34.4) | 0.006 |
| Clinical presentation |  |  |  | <0.001 |
| STEMI, n (%) | 618 (24.5) | 241 (22.7) | 377 (25.8) |  |
| NSTE-ACS, n (%) | 1492 (59.2) | 583 (54.9) | 909 (62.2) |  |
| SA, n (%) | 412 (16.3) | 237 (22.3) | 175 (12.0) |  |
| Laboratory data |  |  |  |  |
| Glycemia, mmol/L | 6.04 ± 3.08 | 5.91 ± 3.61 | 6.10 ± 2.76 | 0.158 |
| Creatinine, μmol/L | 72.53 ± 34.03 | 72.82 ± 39.63 | 72.36 ± 30.25 | 0.756 |
| Uric acid, μmol/L | 303.18 ± 93.17 | 299.10 ± 85.49 | 305.60 ± 97.38 | 0.107 |
| TG, mmol/L | 1.57 (1.14, 2.25) | 1.49 (1.09, 2.15) | 1.61 (1.16, 2.33) | 0.004 |
| TC, mmol/L | 4.26 ± 1.06 | 4.18 ± 1.04 | 4.30 ± 1.08 | 0.010 |
| HDL-C, mmol/L | 1.06 ± 0.32 | 1.02 ± 0.27 | 1.08 ± 0.34 | <0.001 |
| LDL-C, mmol/L | 2.67 ± 0.94 | 2.55 ± 0.89 | 2.74 ± 0.95 | <0.001 |
| Treatment |  |  |  |  |
| Aspirin, n (%) | 2487 (98.7) | 1049 (98.9) | 1438 (98.6) | 0.501 |
| Clopidogrel, n (%) | 2420 (96.1) | 1008 (95.3) | 1412 (96.7) | 0.052 |
| Beta blocker, n (%) | 1718 (68.1) | 701 (66.1) | 1017 (69.6) | 0.060 |
| ACEI, n (%) | 1354 (53.7) | 500 (47.1) | 854 (58.5) | <0.001 |
| CCB, n (%) | 598 (23.7) | 238 (22.4) | 360 (24.6) | 0.198 |
| Statin, n (%) | 2296 (91.0) | 931 (87.7) | 1365 (93.4) | <0.001 |
| Number of diseased vessels |  |  |  | 0.060 |
| 1-vessel disease, n (%) | 987 (39.3) | 442 (42.0) | 545 (37.3) |  |
| 2-vessel disease, n (%) | 926 (36.8) | 372 (35.3) | 554 (37.9) |  |
| 3-vessel disease, n (%) | 601 (23.9) | 239 (22.7) | 362 (24.8) |  |
| Location of target lesions |  |  |  |  |
| LM, n (%) | 85 (3.4) | 39 (3.7) | 46 (3.1) | 0.469 |
| LAD, n (%) | 2084 (82.6) | 863 (81.3) | 1221 (83.6) | 0.144 |
| LCX, n (%) | 1218 (48.3) | 501 (47.2) | 717 (49.1) | 0.357 |
| RCA, n (%) | 1249 (49.5) | 513 (48.4) | 736 (50.4) | 0.315 |
| Characteristics of lesions |  |  |  |  |
| Occlusion, n (%) | 329 (13.0) | 132 (12.4) | 197 (13.5) | 0.443 |
| CTO, n (%) | 226 (9.0) | 105 (9.9) | 121 (8.3) | 0.161 |
| Ostial lesion, n (%) | 274 (10.9) | 104 (9.8) | 170 (11.6) | 0.144 |
| Bifurcation lesion, n (%) | 445 (17.6) | 194 (18.3) | 251 (17.2) | 0.473 |
| Number of treated vessels |  |  |  | 0.573 |
| 1-vessel disease, n (%) | 1454 (57.7) | 601 (56.6) | 853 (58.4) |  |
| 2-vessel disease, n (%) | 838 (33.2) | 357 (33.6) | 481 (32.9) |  |
| ≥3-vessel disease, n (%) | 230 (9.1) | 103 (9.7) | 127 (8.7) |  |
| Length of stents, (mm) | 50.12 ± 32.61 | 52.28 ± 34.33 | 48.55 ± 31.24 | 0.005 |
| Diameter of stents, (mm) | 3.10 ± 0.87 | 3.06 ± 0.43 | 3.12 ± 1.09 | 0.095 |
| MACCEs, n (%) | 422 (16.7) | 227 (21.4) | 195 (13.3) | <0.001 |

ACEI, angiotensin converting enzyme inhibitor; AMI, acute myocardial infraction; BMI, body mass index; CCB, calcium channel blocker; CTO, chronic total occlusions; HDL-C, high-density lipoprotein cholesterol; LAD, left anterior descending; LCX, left circumflex artery; LDL-C, low-density lipoprotein cholesterol; LM, left main coronary artery; MACCEs, major adverse cardiac and cerebrovascular events; METS-IR, metabolic score for insulin resistance; NSTEMI, ono-ST elevation myocardial infarction; PCI, percutaneous coronary intervention; RCA, right coronary artery; SA, stable angina; STEMI, ST elevation myocardial infarction; TC, total cholesterol; TG, triglyceride; TG/HDL-C, triglyceride to high-density lipoprotein cholesterol ratio; TyG, triglyceride and glucose; TyG-BMI, triglyceride glucose-body mass index

**Table S3** Baseline characteristics of participants by age

| **Characteristics** | **Overall**  **(n=1461)** | **<60 years**  **(n=656)** | **≥60 years**  **(n=805)** | ***P* value** |
| --- | --- | --- | --- | --- |
| Demographics |  |  |  |  |
| Age, years | 60.10 ± 11.11 | 50.10 ± 6.83 | 68.25 ± 6.15 | <0.001 |
| Female, (%) | 459 (31.4) | 139 (21.2) | 320 (39.8) | <0.001 |
| BMI, kg/m^2^ | 23.85 ± 3.80 | 23.44 ± 3.72 | 24.19 ± 3.84 | <0.001 |
| Medical history |  |  |  |  |
| Heart failure, n (%) | 161 (11.0) | 60 (9.2) | 101 (12.5) | 0.041 |
| Atrial fibrillation, n (%) | 25 (1.7) | 4 (0.6) | 21 (2.6) | 0.003 |
| Previous AMI, n (%) | 130 (8.9) | 51 (7.8) | 79 (9.8) | 0.173 |
| Previous stroke, n (%) | 64 (4.4) | 22 (3.4) | 42 (5.2) | 0.083 |
| Previous PCI, n (%) | 82 (5.6) | 31 (4.7) | 51 (6.3) | 0.186 |
| Hypertension, n (%) | 726 (49.7) | 304 (46.3) | 422 (52.5) | 0.019 |
| Diabetes mellitus, n (%) | 329 (22.5) | 118 (18.0) | 211 (26.2) | <0.001 |
| Smoking, n (%) | 502 (34.4) | 282 (43.0) | 220 (27.3) | <0.001 |
| Clinical presentation |  |  |  | 0.002 |
| STEMI, n (%) | 377 (25.8) | 195 (29.7) | 182 (22.6) |  |
| NSTE-ACS, n (%) | 909 (62.2) | 397 (60.5) | 512 (63.60) |  |
| SA, n (%) | 175 (12.0) | 64 (9.8) | 111 (13.8) |  |
| Laboratory data |  |  |  |  |
| Glycemia, mmol/L | 6.10 ± 2.76 | 6.10 ± 3.09 | 6.11 ± 2.45 | 0.914 |
| Creatinine, μmol/L | 72.36 ± 30.25 | 70.58 ± 27.98 | 73.81 ± 31.93 | 0.044 |
| Uric acid, μmol/L | 305.60 ± 97.38 | 305.31 ± 88.10 | 305.83 ± 104.38 | 0.919 |
| TG, mmol/L | 1.61 (1.16, 2.33) | 1.78 (1.26, 2.48) | 1.51 (1.10, 2.17) | <0.001 |
| TC, mmol/L | 4.30 ± 1.08 | 4.32 ± 1.14 | 4.29 ± 1.02 | 0.606 |
| HDL-C, mmol/L | 1.08 ± 0.34 | 1.04 ± 0.32 | 1.12 ± 0.35 | <0.001 |
| LDL-C, mmol/L | 2.74 ± 0.95 | 2.75 ± 0.99 | 2.73 ± 0.92 | 0.622 |
| Treatment |  |  |  |  |
| Aspirin, n (%) | 1438 (98.6) | 646 (98.6) | 792 (98.5) | 0.850 |
| Clopidogrel, n (%) | 1412 (96.6) | 638 (97.3) | 774 (96.1) | 0.496 |
| Beta blocker, n (%) | 1017 (69.6) | 480 (73.2) | 537 (66.7) | 0.008 |
| ACEI, n (%) | 854 (58.5) | 399 (60.8) | 455 (56.6) | 0.103 |
| CCB, n (%) | 360 (24.6) | 158 (24.1) | 202 (25.1) | 0.657 |
| Statin, n (%) | 1365 (93.4) | 610 (93.0) | 755 (93.8) | 0.539 |
| Number of diseased vessels |  |  |  | <0.001 |
| 1-vessel disease, n (%) | 545 (37.3) | 306 (46.6) | 239 (29.7) |  |
| 2-vessel disease, n (%) | 554 (37.9) | 230 (35.1) | 324 (40.2) |  |
| 3-vessel disease, n (%) | 362 (24.8) | 120 (18.3) | 242 (30.1) |  |
| Location of target lesions |  |  |  |  |
| LM, n (%) | 46 (3.1) | 13 (2.0) | 33 (4.1) | 0.021 |
| LAD, n (%) | 1221 (83.6) | 532 (81.1) | 689 (85.6) | 0.021 |
| LCX, n (%) | 717 (49.1) | 287 (43.8) | 430 (53.4) | <0.001 |
| RCA, n (%) | 736 (50.4) | 286 (43.6) | 450 (55.9) | <0.001 |
| Characteristics of lesions |  |  |  |  |
| Occlusion, n (%) | 197 (13.5) | 96 (14.6) | 101 (12.5) | 0.245 |
| CTO, n (%) | 121 (8.3) | 44 (6.7) | 77 (9.6) | 0.049 |
| Ostial lesion, n (%) | 170 (11.6) | 73 (11.1) | 97 (12.0) | 0.585 |
| Bifurcation lesion, n (%) | 251 (17.2) | 111 (16.9) | 140 (17.4) | 0.813 |
| Number of treated vessels |  |  |  | <0.001 |
| 1-vessel disease, n (%) | 853 (58.4) | 422 (64.3) | 431 (53.5) |  |
| 2-vessel disease, n (%) | 481 (32.9) | 192 (29.3) | 289 (35.9) |  |
| ≥3-vessel disease, n (%) | 127 (8.7) | 42 (6.4) | 85 (10.6) |  |
| Length of stents, (mm) | 48.55 ± 31.24 | 44.92 ± 30.72 | 51.51 ± 31.36 | <0.001 |
| Diameter of stents, (mm) | 3.12 ± 1.09 | 3.19 ± 1.15 | 3.06 ± 1.03 | 0.022 |
| TyG index | 8.94 ± 0.67 | 9.01 ± 0.67 | 8.89 ± 0.66 | 0.001 |
| TyG-BMI index | 213.54 ± 38.93 | 211.31 ± 37.83 | 215.36 ± 39.74 | 0.048 |
| TG/HDL-C ratio | 3.65 (2.37, 5.67) | 3.98 (2.65, 6.42) | 3.36 (2.16, 5.18) | <0.001 |
| METS-IR | 38.53 ± 7.73 | 38.51 ± 7.97 | 38.55 ± 7.54 | 0.911 |
| MACCEs, n (%) | 195 (13.3) | 58 (8.8) | 137 (17.0) | <0.001 |

ACEI, angiotensin converting enzyme inhibitor; AMI, acute myocardial infraction; BMI, body mass index; CCB, calcium channel blocker; CTO, chronic total occlusions; HDL-C, high-density lipoprotein cholesterol; LAD, left anterior descending; LCX, left circumflex artery; LDL-C, low-density lipoprotein cholesterol; LM, left main coronary artery; MACCEs, major adverse cardiac and cerebrovascular events; METS-IR, metabolic score for insulin resistance; NSTEMI, ono-ST elevation myocardial infarction; PCI, percutaneous coronary intervention; RCA, right coronary artery; SA, stable angina; STEMI, ST elevation myocardial infarction; TC, total cholesterol; TG, triglyceride; TG/HDL-C, triglyceride to high-density lipoprotein cholesterol ratio; TyG, triglyceride and glucose; TyG-BMI, triglyceride glucose-body mass index

**Table S4** Baseline characteristics of participants by sex

| **Characteristics** | **Overall**  **(n=1461)** | **Female**  **(n=459)** | **Male**  **(n=1002)** | ***P* value** |
| --- | --- | --- | --- | --- |
| Demographics |  |  |  |  |
| Age, years | 60.10 ± 11.11 | 63.24 ± 10.04 | 58.67 ± 11.28 | <0.001 |
| BMI, kg/m^2^ | 23.85 ± 3.80 | 25.70 ± 3.72 | 23.00 ± 3.53 | <0.001 |
| Medical history |  |  |  |  |
| Heart failure, n (%) | 161 (11.0) | 62 (13.5) | 99 (9.9) | 0.039 |
| Atrial fibrillation, n (%) | 25 (1.7) | 9 (2.0) | 16 (1.6) | 0.619 |
| Previous AMI, n (%) | 130 (8.9) | 25 (5.4) | 105 (10.5) | 0.002 |
| Previous stroke, n (%) | 64 (4.4) | 22 (4.8) | 42 (4.2) | 0.602 |
| Previous PCI, n (%) | 82 (5.6) | 18 (3.9) | 64 (6.4) | 0.057 |
| Hypertension, n (%) | 726 (49.7) | 271 (59.2) | 455 (45.4) | <0.001 |
| Diabetes mellitus, n (%) | 329 (22.5) | 122 (26.6) | 207 (20.7) | 0.011 |
| Smoking, n (%) | 502 (34.4) | 6 (1.3) | 496 (49.5) | <0.001 |
| Clinical presentation |  |  |  | 0.001 |
| STEMI, n (%) | 377 (25.8) | 91 (19.8) | 286 (28.5) |  |
| NSTE-ACS, n (%) | 909 (62.2) | 317 (69.1) | 592 (59.1) |  |
| SA, n (%) | 175 (12.0) | 51 (11.1) | 124 (12.4) |  |
| Laboratory data |  |  |  |  |
| Glycemia, mmol/L | 6.10 ± 2.76 | 6.29 ± 2.72 | 6.02 ± 2.77 | 0.078 |
| Creatinine, μmol/L | 72.36 ± 30.25 | 61.60 ± 32.76 | 77.28 ± 27.69 | <0.001 |
| Uric acid, μmol/L | 305.60 ± 97.38 | 274.97 ± 104.93 | 319.70 ± 90.33 | <0.001 |
| TG, mmol/L | 1.61 (1.16, 2.33) | 1.65 (1.18, 2.38) | 1.60 (1.16, 2.29) | 0.628 |
| TC, mmol/L | 4.30 ± 1.08 | 4.47 ± 1.04 | 4.23 ± 1.08 | <0.001 |
| HDL-C, mmol/L | 1.08 ± 0.34 | 1.17 ± 0.35 | 1.05 ± 0.33 | <0.001 |
| LDL-C, mmol/L | 2.74 ± 0.95 | 2.84 ± 1.00 | 2.69 ± 0.92 | 0.004 |
| Treatment |  |  |  |  |
| Aspirin, n (%) | 1438 (98.6) | 447 (97.4) | 991 (99.1) | 0.011 |
| Clopidogrel, n (%) | 1412 (96.6) | 452 (98.5) | 958 (95.8) | 0.030 |
| Beta blocker, n (%) | 1017 (69.6) | 320 (69.7) | 697 (69.6) | 0.952 |
| ACEI, n (%) | 854 (58.5) | 259 (56.4) | 595 (59.4) | 0.278 |
| CCB, n (%) | 360 (24.6) | 132 (28.8) | 228 (22.8) | 0.013 |
| Statin, n (%) | 1365 (93.4) | 428 (93.2) | 937 (93.5) | 0.848 |
| Number of diseased vessels |  |  |  | 0.757 |
| 1-vessel disease, n (%) | 545 (37.3) | 165 (35.9) | 380 (37.9) |  |
| 2-vessel disease, n (%) | 554 (37.9) | 179 (39.0) | 375 (37.4) |  |
| 3-vessel disease, n (%) | 362 (24.8) | 115 (25.1) | 247 (24.7) |  |
| Location of target lesions |  |  |  |  |
| LM, n (%) | 46 (3.1) | 13 (2.8) | 33 (3.3) | 0.639 |
| LAD, n (%) | 1221 (83.6) | 385 (83.9) | 836 (83.4) | 0.831 |
| LCX, n (%) | 717 (49.1) | 212 (46.2) | 505 (50.4) | 0.135 |
| RCA, n (%) | 736 (50.4) | 246 (53.6) | 490 (48.9) | 0.096 |
| Characteristics of lesions |  |  |  |  |
| Occlusion, n (%) | 197 (13.5) | 46 (10.0) | 151 (15.1) | 0.009 |
| CTO, n (%) | 121 (8.3) | 37 (8.1) | 84 (8.4) | 0.836 |
| Ostial lesion, n (%) | 170 (11.6) | 49 (10.7) | 121 (12.1) | 0.438 |
| Bifurcation lesion, n (%) | 251 (17.2) | 78 (17.0) | 173 (17.3) | 0.898 |
| Number of treated vessels |  |  |  | 0.494 |
| 1-vessel disease, n (%) | 853 (58.4) | 259 (56.4) | 594 (59.3) |  |
| 2-vessel disease, n (%) | 481 (32.9) | 161 (35.1) | 320 (31.9) |  |
| ≥3-vessel disease, n (%) | 127 (8.7) | 39 (8.5) | 88 (8.8) |  |
| Length of stents, (mm) | 48.55 ± 31.24 | 48.40 ± 31.13 | 48.62 ± 31.31 | 0.902 |
| Diameter of stents, (mm) | 3.12 ± 1.09 | 3.01 ± 0.43 | 3.17 ± 1.28 | 0.012 |
| TyG index | 8.94 ± 0.67 | 8.98 ± 0.66 | 8.93 ± 0.67 | 0.238 |
| TyG-BMI index | 213.54 ± 38.93 | 230.92 ± 39.33 | 205.58 ± 36.07 | <0.001 |
| TG/HDL-C ratio | 3.65 (2.37, 5.67) | 3.41 (2.16, 5.31) | 3.73 (2.46, 5.85) | 0.007 |
| METS-IR | 38.53 ± 7.73 | 40.87 ± 7.86 | 37.46 ± 7.43 | <0.001 |
| MACCEs, n (%) | 195 (13.3) | 64 (13.9) | 131 (13.1) | 0.650 |

ACEI, angiotensin converting enzyme inhibitor; AMI, acute myocardial infraction; BMI, body mass index; CCB, calcium channel blocker; CTO, chronic total occlusions; HDL-C, high-density lipoprotein cholesterol; LAD, left anterior descending; LCX, left circumflex artery; LDL-C, low-density lipoprotein cholesterol; LM, left main coronary artery; MACCEs, major adverse cardiac and cerebrovascular events; METS-IR, metabolic score for insulin resistance; NSTEMI, ono-ST elevation myocardial infarction; PCI, percutaneous coronary intervention; RCA, right coronary artery; SA, stable angina; STEMI, ST elevation myocardial infarction; TC, total cholesterol; TG, triglyceride; TG/HDL-C, triglyceride to high-density lipoprotein cholesterol ratio; TyG, triglyceride and glucose; TyG-BMI, triglyceride glucose-body mass index

**Table S5** Association between MACCEs and risk factor in in overall population

| Variables | B | OR | | *P* value |
| --- | --- | --- | --- | --- |
| Age, years | 0.046 | 1.05 (1.03–1.06) | **<0.001** | |
| Female, (%) | 0.074 | 1.08 (0.78–1.49) | 0.650 | |
| BMI, kg/m2 | 0.033 | 1.03 (0.99–1.07) | 0.102 | |
| Heart failure, n (%) | 0.547 | 1.73 (1.13–2.63) | **0.011** | |
| Atrial fibrillation, n (%) | 0.216 | 1.24 (0.42–3.66) | 0.695 | |
| Previous AMI, n (%) | 0.596 | 1.82 (1.15–2.86) | **0.010** | |
| Previous stroke, n (%) | 0.063 | 1.07 (0.52–2.19) | 0.863 | |
| Previous PCI, n (%) | 0.309 | 1.36 (0.75–2.47) | 0.310 | |
| Hypertension, n (%) | 0.310 | 1.36 (1.01–1.85) | **0.045** | |
| Diabetes mellitus, n (%) | 0.535 | 1.71 (1.23–2.38) | **0.002** | |
| Smoking, n (%) | 0.129 | 1.14 (0.83–1.56) | 0.418 | |
| Clinical presentation | 0.016 | 1.02 (0.79–1.31) | 0.902 | |
| Glycemia, mmol/L | 0.001 | 1.00 (0.95–1.06) | 0.963 | |
| Creatinine, μmol/L | 0.006 | 1.01 (1.00–1.01) | **0.005** | |
| Uric acid, μmol/L | 0.002 | 1.00 (1.00–1.00) | **0.026** | |
| TG, mmol/L | 0.019 | 1.02 (0.92–1.13) | 0.703 | |
| TC, mmol/L | 0.083 | 1.09 (0.95–1.25) | 0.232 | |
| HDL-C, mmol/L | 0.114 | 1.12 (0.72–1.74) | 0.609 | |
| LDL-C, mmol/L | 0.132 | 1.14 (0.98–1.33) | 0.092 | |
| Clopidogrel, n (%) | 0.064 | 1.07 (0.52–2.20) | 0.862 | |
| Beta blocker, n (%) | 0.270 | 1.31 (0.93–1.85) | 0.122 | |
| ACEI, n (%) | 0.675 | 1.96 (1.41–2.74) | **<0.001** | |
| CCB, n (%) | 0.061 | 1.06 (0.75–1.50) | 0.728 | |
| Statin, n (%) | 0.708 | 2.03 (0.93–4.45) | 0.077 | |
| Number of diseased vessels | 0.501 | 1.65 (1.36–2.01) | **<0.001** | |
| LM, n (%) | 0.613 | 1.85 (0.90–3.79) | 0.094 | |
| LAD, n (%) | 0.610 | 1.84 (1.13–2.99) | **0.014** | |
| LCX, n (%) | 0.292 | 1.34 (0.99–1.81) | 0.059 | |
| RCA, n (%) | 0.574 | 1.78 (1.30–2.42) | **<0.001** | |
| Occlusion, n (%) | -0.120 | 0.89 (0.56–1.40) | 0.606 | |
| CTO, n (%) | 1.003 | 2.73 (1.77–4.20) | **<0.001** | |
| Ostial lesion, n (%) | 0.074 | 1.08 (0.68–1.71) | 0.753 | |
| Bifurcation lesion, n (%) | 0.021 | 1.02 (0.69–1.52) | 0.919 | |
| Length of stents, (mm) | 0.010 | 1.01 (1.01–1.01) | **<0.001** | |
| Diameter of stents, (mm) | -0.534 | 0.59 (0.41–0.85) | **0.004** | |
| TyG index | -0.013 | 0.99 (0.79–1.24) | 0.912 | |
| TyG-BMI index | 0.003 | 1.00 (1.00–1.01) | 0.137 | |
| TG/HDL-C ratio | 0.008 | 1.01 (0.98–1.04) | 0.610 | |
| METS-IR | 0.014 | 1.01 (1.00–1.03) | 0.162 | |

**Table S6** Association between MACCEs and risk factor in in elderly patients

| Variables | B | OR | | *P* value |
| --- | --- | --- | --- | --- |
| Age, years | 0.069 | 1.07 (1.04–1.10) | **<0.001** | |
| Female, (%) | 0.056 | 1.06 (0.73–1.54) | 0.768 | |
| BMI, kg/m2 | 0.054 | 1.06 (0.99–1.11) | 0.051 | |
| Heart failure, n (%) | 0.678 | 1.97 (1.21–3.20) | **0.006** | |
| Atrial fibrillation, n (%) | 0.141 | 1.15 (0.38–3.48) | 0.802 | |
| Previous AMI, n (%) | 0.791 | 2.21 (1.30–3.73) | **0.003** | |
| Previous stroke, n (%) | -0.027 | 0.97 (0.42–2.24) | 0.950 | |
| Previous PCI, n (%) | 0.186 | 1.20 (0.59–2.47) | 0.612 | |
| Hypertension, n (%) | 0.252 | 1.29 (0.89–1.87) | 0.183 | |
| Smoking, n (%) | 0.238 | 1.27 (0.85–1.89) | 0.243 | |
| Clinical presentation | -0.072 | 0.93 (0.68–1.27) | 0.647 | |
| Glycemia, mmol/L | 0.007 | 1.01 (0.94–1.08) | 0.861 | |
| Creatinine, μmol/L | 0.007 | 1.01 (1.00–1.01) | **0.005** | |
| Uric acid, μmol/L | 0.002 | 1.00 (1.00–1.00) | **0.019** | |
| TG, mmol/L | -0.014 | 0.99 (0.86–1.14) | 0.843 | |
| TC, mmol/L | 0.039 | 1.04 (0.87–1.24) | 0.67 | |
| HDL-C, mmol/L | -0.051 | 0.95 (0.56–1.62) | 0.851 | |
| LDL-C, mmol/L | 0.115 | 1.12 (0.92–1.37) | 0.26 | |
| Clopidogrel, n (%) | 0.305 | 1.36 (0.57–3.23) | 0.492 | |
| Beta blocker, n (%) | 0.357 | 1.43 (0.95–2.15) | 0.088 | |
| ACEI, n (%) | 0.822 | 2.28 (1.52–3.41) | **<0.001** | |
| CCB, n (%) | 0.029 | 1.03 (0.68–1.57) | 0.893 | |
| Statin, n (%) | 0.900 | 2.46 (0.87–6.95) | 0.090 | |
| Number of diseased vessels | 0.350 | 1.42 (1.11–1.81) | **0.005** | |
| LM, n (%) | -0.411 | 0.66 (0.23–1.92) | 0.448 | |
| LAD, n (%) | 0.759 | 2.14 (1.12–4.09) | **0.022** | |
| LCX, n (%) | 0.064 | 1.07 (0.74–1.54) | 0.732 | |
| RCA, n (%) | 0.494 | 1.64 (1.12–2.41) | **0.012** | |
| Occlusion, n (%) | -0.184 | 0.83 (0.47–1.49) | 0.536 | |
| CTO, n (%) | 0.902 | 2.47 (1.46–4.16) | **0.001** | |
| Ostial lesion, n (%) | -0.129 | 0.88 (0.49–1.58) | 0.664 | |
| Bifurcation lesion, n (%) | -0.180 | 0.84 (0.50–1.38) | 0.485 | |
| Length of stents, (mm) | 0.009 | 1.01 (1.00–1.02) | **0.001** | |
| Diameter of stents, (mm) | -0.183 | 0.83 (0.53–1.31) | 0.425 | |
| TyG index | 0.017 | 1.02 (0.77–1.34) | 0.904 | |
| TyG-BMI index | 0.005 | 1.01 (1.00–1.01) | **0.036** | |
| TG/HDL-C ratio | 0.001 | 1.00 (0.96–1.05) | 0.967 | |
| METS-IR | 0.029 | 1.03 (1.01–1.05) | **0.017** | |

**Table S7** Association between MACCEs and risk factor in in female patients

| Variables | B | OR | | *P* value |
| --- | --- | --- | --- | --- |
| Age, years | 0.082 | 1.09 (1.05–1.12) | **<0.001** | |
| BMI, kg/m2 | 0.059 | 1.06 (0.99–1.14) | 0.091 | |
| Heart failure, n (%) | 0.59 | 1.80 (0.91–3.54) | 0.091 | |
| Atrial fibrillation, n (%) | -0.264 | 0.77 (0.09–6.24) | 0.805 | |
| Previous AMI, n (%) | 0.945 | 2.57 (1.03–6.43) | **0.043** | |
| Previous stroke, n (%) | 0.334 | 1.40 (0.46–4.27) | 0.558 | |
| Previous PCI, n (%) | 0.220 | 1.25 (0.35–4.43) | 0.734 | |
| Hypertension, n (%) | 0.162 | 1.18 (0.68–2.03) | 0.559 | |
| Smoking, n (%) | 1.148 | 3.15 (0.57–17.58) | 0.190 | |
| Clinical presentation | -0.448 | 0.64 (0.39–1.04) | 0.070 | |
| Glycemia, mmol/L | 0.039 | 1.04 (0.95–1.13) | 0.383 | |
| Creatinine, μmol/L | 0.008 | 1.01 (1.00–1.02) | **0.026** | |
| Uric acid, μmol/L | 0.004 | 1.00 (1.00–1.01) | **0.003** | |
| TG, mmol/L | 0.219 | 1.25 (0.98–1.52) | 0.053 | |
| TC, mmol/L | 0.151 | 1.16 (0.91–1.48) | 0.225 | |
| HDL-C, mmol/L | -0.041 | 0.96 (0.45–2.07) | 0.917 | |
| LDL-C, mmol/L | 0.051 | 1.05 (0.81–1.37) | 0.702 | |
| Clopidogrel, n (%) | -0.954 | 0.39 (0.08–1.91) | 0.242 | |
| Beta blocker, n (%) | 0.033 | 1.03 (0.58–1.84) | 0.911 | |
| ACEI, n (%) | 0.695 | 2.00 (1.13–3.55) | **0.017** | |
| CCB, n (%) | -0.221 | 0.80 (0.44–1.47) | 0.475 | |
| Statin, n (%) | 0.899 | 2.46 (0.57–10.56) | 0.227 | |
| Number of diseased vessels | 0.454 | 1.57 (1.12–2.22) | **0.010** | |
| LM, n (%) | 0.119 | 1.13 (0.24–5.20) | 0.879 | |
| LAD, n (%) | 0.509 | 1.66 (0.73–3.81) | 0.228 | |
| LCX, n (%) | 0.032 | 1.03 (0.61–1.75) | 0.905 | |
| RCA, n (%) | 0.827 | 2.29 (1.30–4.05) | **0.005** | |
| Occlusion, n (%) | -0.579 | 0.56 (0.19–1.62) | 0.285 | |
| CTO, n (%) | 0.926 | 2.52 (1.16–5.51) | **0.020** | |
| Ostial lesion, n (%) | 0.032 | 1.03 (0.44–2.41) | 0.942 | |
| Bifurcation lesion, n (%) | -0.116 | 0.89 (0.43–1.84) | 0.753 | |
| Length of stents, (mm) | 0.010 | 1.01 (1.00–1.02) | **0.011** | |
| Diameter of stents, (mm) | -0.680 | 0.51 (0.26–1.00) | 0.050 | |
| TyG index | 0.455 | 1.58 (1.07–2.32) | **0.021** | |
| TyG-BMI index | 0.008 | 1.01 (1.00–1.02) | **0.013** | |
| TG/HDL-C ratio | 0.075 | 1.08 (1.01–1.15) | **0.021** | |
| METS-IR | 0.036 | 1.04 (1.01–1.07) | **0.024** | |
