# Supplementary figures and images for: Association between non-insulin-based insulin resistance indices and cardiovascular events in patients undergoing percutaneous coronary intervention: a retrospective study
Source: Cardiovasc Diabetol. 2023 Jun 29;22:161. doi: 10.1186/s12933-023-01898-1 (PMC10311786; doi:10.1186/s12933-023-01898-1)

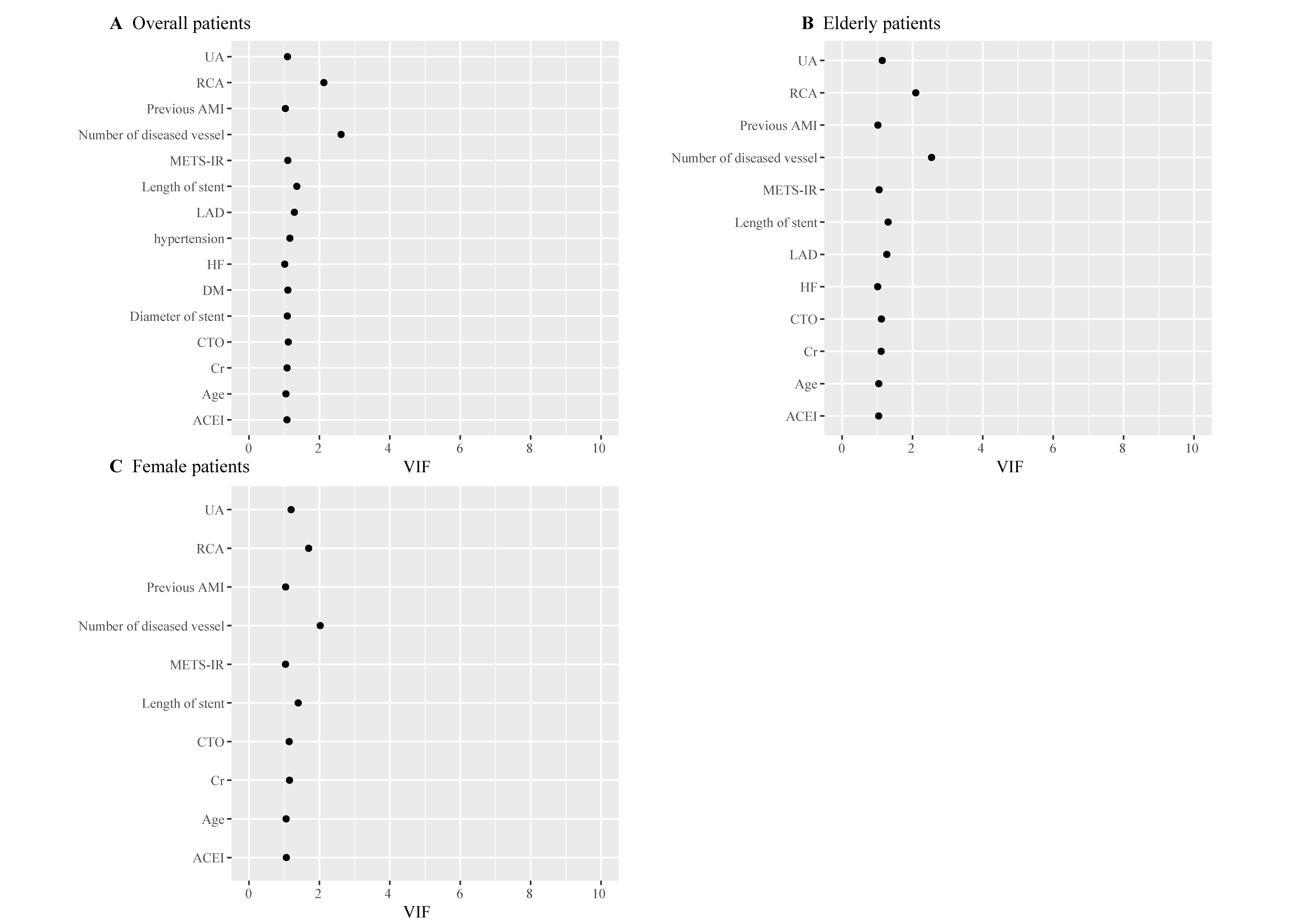

Supplement: Supplementary file 2 — Supplementary Material 2 [file 12933_2023_1898_MOESM2_ESM.tif]
